# Single‐cell transcriptomic analysis deciphers key transitional signatures associated with oncogenic evolution in human intramucosal oesophageal squamous cell carcinoma

**DOI:** 10.1002/ctm2.1203

**Published:** 2023-02-28

**Authors:** Xin‐Yang Liu, Yan‐Bo Liu, Jia‐Cheng Xu, Yi‐Fei Zhang, Yuan‐Yuan Ruan, Yi Zhao, Lin‐Feng Wu, Jian‐Wei Hu, Zhen Zhang, Meng‐Jiang He, Tian‐Yin Chen, Xiao‐Yue Xu, Jing‐Wei Zhang, Yi‐Qun Zhang, Ping‐Hong Zhou

**Affiliations:** ^1^ Department of Endoscopy Center and Endoscopy Research Institute Zhongshan Hospital Fudan University Shanghai China; ^2^ Department of Endoscopy Shanghai Collaborative Innovation Center Shanghai China; ^3^ Department of Biochemistry and Molecular Biology, School of Basic Medical Sciences Fudan University Shanghai China; ^4^ GobioX BioTech Shanghai China; ^5^ Department of Genetic Engineering State Key Laboratory School of Life Sciences Fudan University Shanghai China

**Keywords:** intramucosal, oesophageal squamous cell carcinoma, single‐cell RNA‐seq, tumour microenvironment

## Abstract

**Background and aims:**

The early diagnosis and intervention of oesophageal squamous cell carcinoma (ESCC) are particularly important because of the lack of effective therapies and poor prognosis. Comprehensive research on early ESCC at the single‐cell level is rare due to the need for fresh and high‐quality specimens obtained from ESD. This study aims to systematically describe the cellular atlas of human intramucosal ESCC.

**Methods:**

Five paired samples of intramucosal ESCC, para‐ESCC oesophageal tissues from endoscopically resected specimens and peripheral blood mononuclear cells were adopted for scRNA‐seq analysis. Computational pipeline scMetabolism was applied to quantify the metabolic diversity of single cells.

**Results:**

A total of 164 715 cells were profiled. Epithelial cells exhibited high intra‐tumoural heterogeneity and two evolutionary trajectories during ESCC tumorigenesis initiated from proliferative cells, and then through an intermediate state, to two different terminal states of normally differentiated epithelial cells or malignant cells, respectively. The abundance of CD8^+^ T_EX_s, Tregs and PD1^+^CD4^+^T cells suggested an exhausted and suppressive immune microenvironment. Several genes in immune cells, such as CXCL13, CXCR5 and PADI4, were identified as new biomarkers for poor prognosis. A new subcluster of malignant cells associated with metastasis and angiogenesis that appeared at an early stage compared with progressive ESCC was also identified in this study. Intercellular interaction analysis based on ligand–receptor pairs revealed the subcluster of malignant cells interacting with CAFs via the MDK–NCL pathway, which was verified by cell proliferation assay and IHC. This indicates that the interaction may be an important hallmark in the early change of tumour microenvironment and serves as a sign of CAF activation to stimulate downstream pathways for facilitating tumour invasion.

**Conclusion:**

This study demonstrates the changes of cell subsets and transcriptional levels in human intramucosal ESCC, which may provide unique insights into the development of novel biomarkers and potential intervention strategies.

## 1 INTRODUCTION

Oesophageal squamous cell carcinoma (ESCC) is one of the most common and deadly gastrointestinal malignancies, which is particularly prominent in Asia.^1^, ^2^ Compared with surgery, endoscopic resection and endoscopic ablation, such as photodynamic therapy, cryotherapy and radiofrequency ablation, can be applied to early ESCC confined to oesophageal mucosal and submucosal layers, with comparative efficacy but fewer complications.^3^ In advanced ESCC, more than 50% of patients may not be eligible for surgery upon diagnosis. Even in patients who have received surgery, the 5‐year metastasis and recurrence rate still exceeds 40%, and the 5‐year survival rate cannot reach 25%.^4^ Currently, there is limited progress in target therapy and immunotherapy.^5^, ^6^ Therefore, the early diagnosis and intervention of ESCC are particularly important.

ESCC undergoes a sequence of development ranging from inflammation, hyperplasia, dysplasia and carcinoma in situ to invasive carcinoma. Exploring the underlying mechanisms for the initiation and development of ESCC is extremely important to early detection and precision treatment. Recent whole‐exome or whole‐genome sequencing studies have identified many genomic variations in ESCC.^7^ However, traditional sequencing studies have obtained the overall average characteristics of cell populations, where the characteristics of the dominant cell population are more likely to be explicit, while the characteristics of rare low abundance are often drowned in a vast number of bulk signals. For the tumour microenvironment ecosystem formed by spatiotemporal interaction, single‐cell transcriptome sequencing provides a promising tool for the in‐depth analysis of multiple heterogeneous cells, including malignant cells, immune cells and stromal cells. Using a such novel technique, the immunosuppressive microenvironment in human progressive ESCC has been considered to be responsible for the failure of immuno‐surveillance.^8^ A set of key transitional signatures associated with the oncogenic evolution of epithelial cells and a chronic inflammatory environment for promoting the survival and proliferation of carcinogen‐transformed epithelial cells were also observed in a mouse model mimicking human ESCC development.^9^ Due to the need for high‐quality fresh specimens and the scarcity of tissues obtained from endoscopic submucosal resection (ESD), there is still a lack of comprehensive research on the tumour ecosystem of early ESCC at the single‐cell level.

To understand the tumour ecosystem of intramucosal ESCC, five patients with Tis and T1a stage ESCC were collected. Specimens from ESD were stained with Lugo's iodine to classify unstained tumour tissues and deeply stained paratumour epithelial mucosa.^10^, ^11^ A total of 164 715 cells and single‐cell transcriptome data were generated by single‐cell transcriptome sequencing of tumour tissues, paired paratumour epithelial mucosa and corresponding peripheral blood mononuclear cells (PBMCs). This study aims to systematically describe the transcriptome landscape of intramucosal ESCC and clarify the changes of cell subsets and transcriptional levels associated with the pathogenesis of early ESCC in the tumour microenvironment. This can provide evidence for the development of novel biomarkers and insights for the early intervention or reversal of ESCC.

## 2 MATERIALS AND METHODS

### 2.1 Patients

For scRNA‐seq, patients clinically diagnosed with Tis and T1a stage ESCC were collected from the Department of Endoscopy, Zhongshan Hospital, Fudan University. Specimens from ESD were stained with Lugo's iodine for classification of unstained tumour tissue and deeply stained paratumour epithelial mucosa. A 1 mm× 1 mm sample from the lesion and a 1 mm× 1 mm sample from the paratumour area was taken from each resected specimen in order to minimise the influence on pathological assessment. PBMCs from each patient were also collected. Final pathological diagnosis was confirmed by evaluating the ESD resected specimen. Patients were excluded if the pathological diagnosis did not meet the standard of Tis or T1a stage. Five pairs of samples were included after pathological confirmation.

Another 15 surgically resected ESCC samples at different stages were included for immunohistochemistry (IHC).

### 2.2 Tissue dissociation and cell purification

Tissues were transported in sterile culture dish with 10 mL 1× Dulbecco's phosphate‐buffered saline (DPBS; Thermo Fisher) on ice to remove the residual tissue storage solution, then minced on ice. Intramucosal ESCC and paratumour tissues were dissociated in 0.1% collagenase IV digestive solution (RPMI‐1640 medium (Invitrogen) with 10% foetal bovine serum (FBS; Thermo Fisher) containing collagenase IV (Sigma)) at 37°C with a shaking speed of 50 rpm for about 40 min.We repeatedly collected the dissociated cells at interval of 20 min to increase cell yield and viability. Cell suspensions were filtered using a 40‐μm nylon cell strainer and red blood cells were removed by 1× Red Blood Cell Lysis Solution (Thermo Fisher). Dissociated cells were washed with 1× DPBS containing 2% FBS. Cells were stained with 0.4% Trypan blue (Thermo Fisher) to check the viability on Countess® II Automated Cell Counter(Thermo Fisher).

PBMCs were isolated using lymphocyte‐h (lymphocyte isolation solution; Cedarlane) and red blood cell lysis solution (Thermo Fisher) according to the manufacturer's instructions. Briefly, 2 mL of fresh peripheral blood was collected prior to surgery in an EDTA anticoagulant tube and subsequently layered onto lymphocyte isolation solution. After centrifugation, lymphocytic cells at the lymphocyte isolation solution interface were carefully transferred to a new tube and red blood cell lysis solution was added to the cells. Lymphocytic cells was washed twice with DPBS. Lymphocytic cells were resuspended with sorting buffer.

### 2.3 Single‐cell RNA sequecing

Following tissue digestion, samples were sequenced by 10× chromium single‐cell platform (10× Genomics, 30 v3 chemistry, Rev C). In detail, Gel bead in EMulsion (GEM) were generated and mRNAs from single cells were then subject to second‐strand cDNA synthesis, adaptor ligation and universal amplification was performed. The read length of sequencing was 150 bp(paired), and log transformation was used for normalisation.Then, all the remaining procedures including the library construction were performed according to the standard manufacturer's protocol (CG000206 RevD). The libraries were sequenced on NovaSeq6000 (Illumina). Single‐cell RNA‐seq data processing reads, alignment and initial clustering of the raw scRNA‐seq profiles were progressive using the Cell Ranger 3.0.1 pipeline with default and recommended parameters.

### 2.4 Data analysis

This output was then imported into the Seurat (4.0.1) R toolkit for quality control and downstream analysis of our scRNA‐seq data. All functions were run with default parameters, unless specified otherwise. We excluded cells with fewer than 200 or more than 6000 detected genes (where each gene had to have at least one UMI aligned in at least three cells). The expression of mitochondria genes was calculated using PercentageFeatureSet function of the Seurat package. To remove low activity cells, cells with more than 10% expression of mitochondria genes were excluded. The normalised data (NormalizeData function in Seurat package) was performed for extracting a subset of variable genes. Variable genes were identified while controlling for the strong relationship between variability and average expression. Next, we integrated data from different samples after identifying ‘anchors’ between datasets using FindIntegrationAnchors and IntegrateData in the seurat package . Then we performed principal component analysis (PCA) and reduced the data to the top 30 PCA components after scaled the data. We visualised the clusters on a 2D map produced with t‐distributed stochastic neighbour embedding (t‐SNE). For sub‐clustering, we applied the same procedure of scaled, dimensionality reduction and clustering to the specific set of data (usually restricted to one type of cell). For each cluster, we used the Wilcoxon Rank‐Sum Test to find significant deferentially expressed genes comparing the remaining clusters. Cells with mix features were removed from further analysis. Clusters were identified using FindClusters function, and the specific gene markers for each cluster were determined using the FindAllMarkers function implanted in Seurat package.

### 2.5 Estimation of the copy number variations

The R package ‘infercnv’^12^ was used to estimate the initial copy number variations (CNVs) in each region. The expression level of each cell acted as the input file with recommendatory parameters. Fibroblasts and endothelial cells served as the background to calculate the CNVs score. The CNV value of each cell was calculated as the quadratic sum of the CNV region. Also, we applied the R package ‘inferCNV, v1.6.0’ to calculate CNVs in tumour cells as described previously.^13^, ^14^

### 2.6 Analyses of metabolic pathways

A recently developed computational pipeline for quantifying single‐cell metabolism, scMetabolism(v0.2.1), was also applied for visualisation and quantifying the metabolic diversity of single cells in each cluster.^15^ ScMetabolism used vision algorithm to score each cluster, and finally obtained the activity score of clusters in each metabolic pathway based on the conventional single‐cell matrix file.

### 2.7 Cell developmental trajectory

The cell lineage trajectory of epithelial cells was inferred by using Slingshot (Street et al. 2017, https://bioconductor.org/packages/devel/bioc/vignettes/slingshot/inst/doc/vignette.html).

### 2.8 Functional enrichment analyses

Gene ontology (GO) and Kyoto Encyclopedia of Genes and Genomes (KEGG) pathway analyses were performed via clusterProfiler(3.18.1),^16^ GSVA(1.38.2) and GSEABase(1.52.1). Notably, *p* < .01 and the number of enriched genes >3 acted as the thresholds for a significant difference in GO or KEGG pathways.

### 2.9 Construction of a cell to cell interaction network

To investigate cell‐to‐cell interaction among T cells, CAFs and epithelial cells, R package ‘CellChat’ (1.1.2)^17^ was applied. The ligand–receptor pairs in CellChat were retrieved from previous studies.^18^

### 2.10 Gene Expression Profiling Interactive Analysis dataset

Gene Expression Profiling Interactive Analysis (GEPIA) uses standard processing pipelines to analyse the RNA sequencing expression data from 9736 tumours and 8587 normal samples from the TCGA and GTEx projects. GEPIA provides customisable features such as dimension reduction analysis, correlation analysis, tumour/normal differential expression analysis, analysis based on cancer types or pathological stages, similar gene detection and patient survival analysis (GEPIA: a web server for cancer and normal gene expression profiling and interactive analyses).

### 2.11 Immunohistochemistry

IHC was performed in ESCC specimen of paratumour tissue, high grade dysplasia, intramucosal ESCC and progressive ESCC. Samples were used for staining of LUM (Cat#ab168348; Abcam) NCL (Cat#ab129200; Abcam) and MDK (Cat#ab52637; Abcam) antibodies. All the staining process was carried out on the IHC/ISH System (BenchMark GX, Roche) following the manufacturer's instruction. Multiplexed IHC (mIHC) was performed using a multiple fluorescent immunohistochemical staining kit (abs50012; Absin, Shanghai, China) according to the manufacturer's instructions.^19^ Sections were observed using an optical microscope (BX53; Olympus, Tokyo, Japan)

### 2.12 Cell culture and transfection

ESCC cell lines KYSE510, KYSE150 and ECA109 were purchased from Cell Bank of Type Culture Collection of Chinese Academy of Sciences. Oesophageal cancer‐associated fibroblasts (CAFs) HUM‐iCELL‐d042 was purchased from iCell (iCell Bioscience Inc, Shanghai). Cancer cells were grown in RPMI 1640 (Gibco, Sigma–Aldrich, USA) supplemented with 10% FBS (Gibco, Sigma–Aldrich) and cultured in 5% CO_2_ at 37°C; and CAFs were grown in ICell Primary Fibroblast Culture System (Cat: PriMed‐iCELL‐003).

Cells were seeded in six‐well plates, and the medium was replaced with Opti‐MEM. Small interfering RNA (siRNA; GenePharma, China) and Lipofectamine 3000 transfection reagent (Life Technologies, USA) were transfected into the cells according to the manufacturer's recommendations. The target siRNA sequence list as follows: NCL siRNA‐1: 5′‐UCCAAGGUAACUUUAUUUCUU‐3′; NCL siRNA‐2: 5′‐UUCUUUGACAGGCUCUUCCUU‐3′.

### 2.13 Western blot analysis

Cells were lysed in RIPA buffer (Cell Signaling, USA) and quantified using the BCA protein assay (Beyotime, Shanghai, China). Equal amounts of protein were subjected to electrophoresis on SDS‐PAGE and were probed with the antibody NCL (Cat: ab129200) purchased from Abcam. The protein bands were measured using a Gel Doc 2000 (Bio‐Rad).

### 2.14 Cell proliferation assays

Approximately 4000 HUM‐iCELL‐d042 cells were plated in 96‐well plates and cultured for 24 h. Cell viability was assessed using Cell Counting Kit‐8 (Dojindo, Kumamoto, Japan) according to the manufacturer's instructions.

### 2.15 Statistical analyses

Cell distribution comparisons between two groups were performed using unpaired two‐tailed Wilcoxon rank‐sum tests. Comparisons of gene expression or gene signature between two groups of cells were performed using unpaired two‐tailed Student's *t*‐test. All statistical analyses and presentation were performed using R and statistical significance was set at *p* < .05.

## 3 RESULTS

### 3.1 Twelve distinct cell populations and differentiation relations identified by the scRNA‐Seq census of human intramucosal ESCC

**FIGURE 1:**
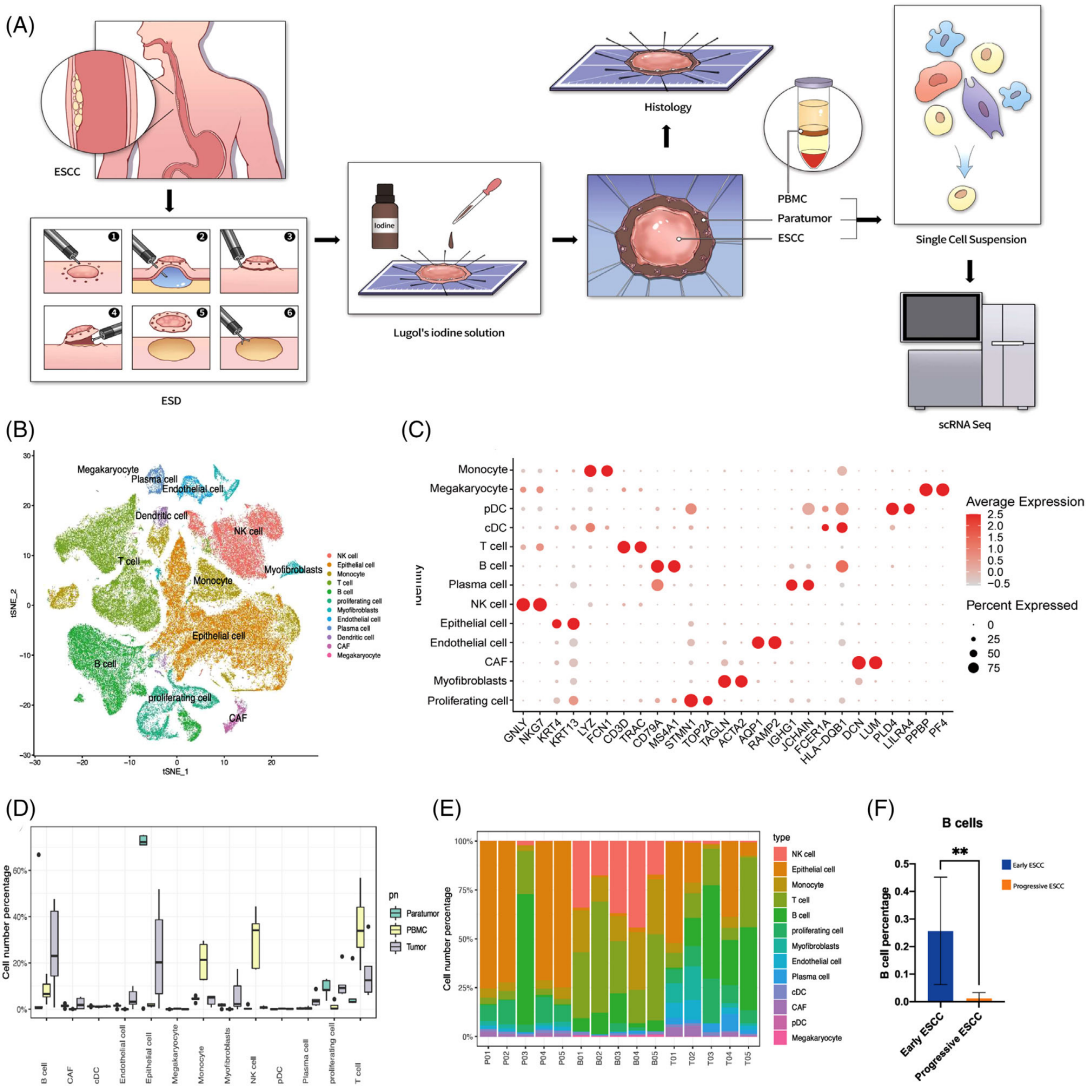
scRNA‐Seq profiling of the human intramucosal ESCC microenvironment. (A) Workflow depicting sample collection, single‐cell preparation and RNA‐seq. (B) t‐SNE plot displaying main cell types in human intramucosal ESCC microenvironment. (C) Bubblemap displaying the top 10 SDE genes in each cell type, both colour and size indicate the effect size. (D) Distribution of cells in different specimens. (E) Distribution of cells in different patients. (F) B cell percentage in early and progressive ESCC patients (^**^ indicates a *p* value < .01). ESCC: oesophageal squamous cell carcinoma; ESD: endoscopic submucosal resection; PBMCs: peripheral blood mononuclear cells; t‐SNE: t‐distributed stochastic neighbour embedding.

To create a comprehensive single‐cell landscape of human intramucosal ESCC, scRNA‐seq was applied using paired samples of endoscopically resected intramucosal ESCC, para‐ESCC oesophageal tissues from endoscopically resected specimens, and PBMCs. Intramucosal ESCC lesions and para‐ESCC oesophageal tissue were identified by iodine staining on the endoscopically resected specimen. The non‐staining area indicated early ESCC, whereas the deep‐staining area represented non‐ESCC paratumour tissue. A 1 mm × 1 mm sample from the lesion and a 1 mm × 1 mm sample from the paratumour area were taken from each resected specimen to minimise the influence on pathological assessment (Figure 1A). Five pairs of samples were included after pathological confirmation, and demographic data are shown in Table 1. After quality control to remove low‐quality cells, a total of 164 715 cells were retained for further analysis. Among them, 48 711 cells derived from intramucosal ESCC samples (6736–11 794, median: 9912/patient), 55 191 cells derived from paratumour samples (8417–13 876, median: 10 127/patient) and 60 813 cells derived from PBMCs (10 422–14 256, median: 11 951/patient). A median of 1634 genes per cell was detected.

**TABLE 1:** Demographic and pathological characteristics of patients.

	Age	Sex	Tissue size (cm)	Iodine uncoloured area (cm)	Microscopic lesion (cm)	Stage (depth of tumour invasion)
Patient0	55	Male	3 × 2.5	2.5 × 1.5	3 × 2	T1a (Muscularis mucosae)
Patient02	65	Female	3.8 × 2.3	2.5 × 1.3	3 × l.2	T1a (Lamina propria mucosa)
Patient03	72	Male	3.9 × 5	2.7 × 2	3 × 2.5	Tis
Patient04	75	Male	9 × 8	8 × 7	4.8 × 4.3	T1a (Lamina propria mucosa)
Patient05	68	Male	5.8 × 2.8	5.2 × 2.2	4.8 × 1.5	T1a (Lamina propria mucosa)

Based on the expression of canonical markers, cells were classified into 12 clusters by using t‐SNE (Figures 1B and S1B), including megakaryocyte cells, CAFs, dendritic cells, plasma cells, endothelial cells, myofibroblasts, proliferating cells, B cells, T cells, monocytes, epithelial cells and natural killer cells. Marker genes of the identified 12 major cell types were cell type specific and consistent with classic, well‐established markers for each cell population (Figures 1C and S1A).

Notably, the proportion of immune cells in the tumour tissue increased significantly compared with the paratumour tissue, such as B and T cells. In contrast, epithelial cells represented the dominant cluster in the paratumour tissue (Figure 1D). More importantly, an increase in the proportion of B cells was observed in both the tumour and paratumour tissues from patient 3 (Figure 1E), who was identified as high‐grade intraepithelial neoplasia rather than carcinoma in situ (Table 1). In intramucosal ESCC, the average proportion of infiltrating B cells was significantly higher than that in progressive ESCC^8^ (Figure 1F). However, a decrease in the proportion of infiltrating B cells in progressive ESCC compared with other cancer types was reported in previous studies.^20^

### 3.2 Transcriptomic inter‐tumour heterogeneity and transitional cell status of epithelial cells in human intramucosal ESCC

**FIGURE 2:**
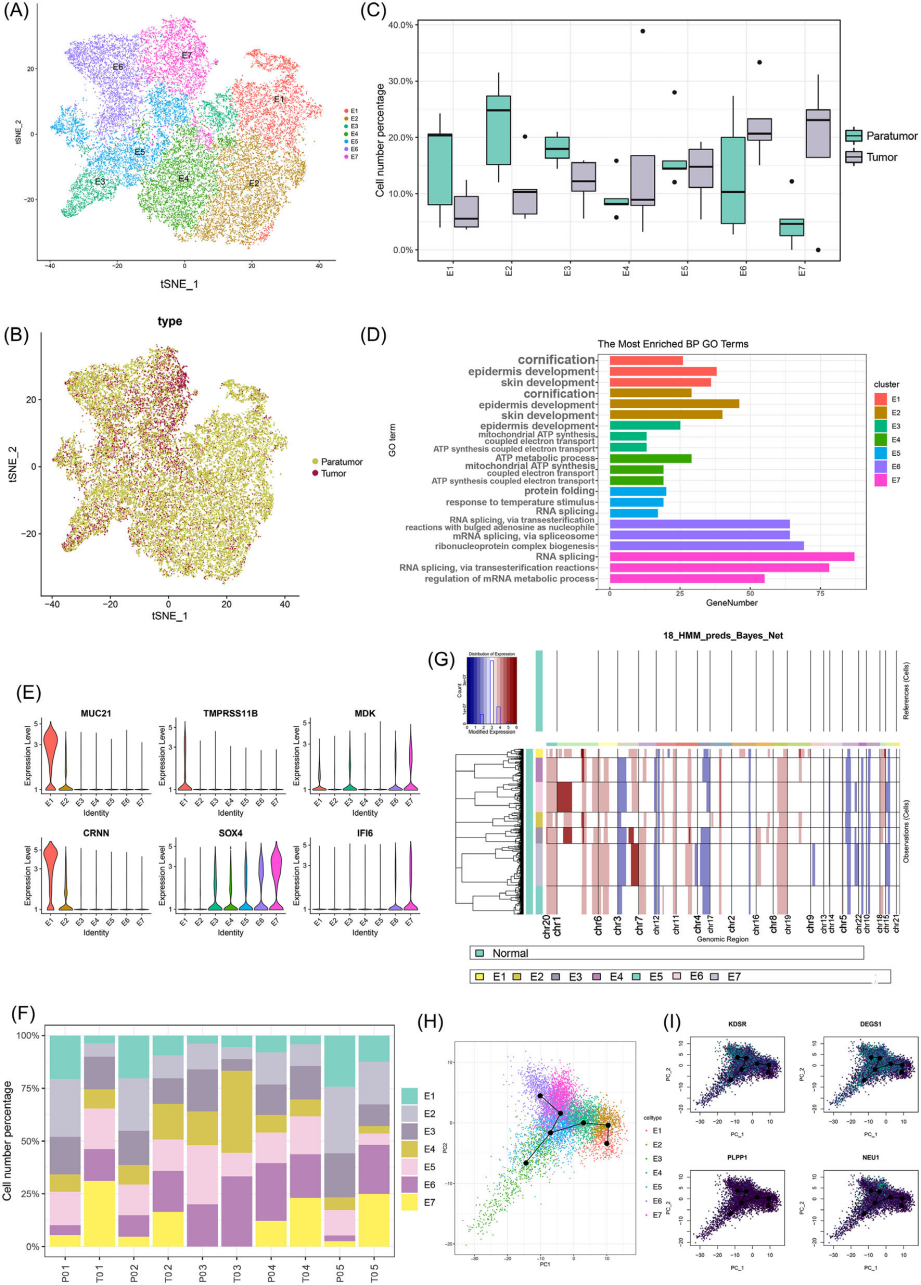
The single‐cell transcriptomes of epithelial cells in human intramucosal ESCC. (A) t‐SNE plot revealing the seven epithelial cell subclusters. (B) Distribution of epithelial cells in paratumour and tumour specimen revealed by t‐SNE plot. (C) Cell number percentage of seven subclusters in different specimen. (D) GO analysis for E1–E7 subclusters. (E) Violin plot showing important markers of E1–E7 subclusters. (F) Distribution of seven subclusters in different patients, with E7 absent in patient 3. (G) CNV profiles showing a relative variation between epithelial cells originated from tumour and paratumour specimen after Bayes normalisation. (H) Psudotome analysis revealing trajectory paths of intramucosal ESCC initiation. (I) t‐SNE plot showing gene expression in sphingolipid metabolism pathways of E7 subcluster. ESCC: oesophageal squamous cell carcinoma; t‐SNE: t‐distributed stochastic neighbour embedding; GO: Gene Ontology; CNV: copy number variation.

To investigate how normal oesophageal epithelium develops into carcinoma, various tools were introduced to evaluate genetic alterations and functional changes in epithelial cells harvested from tumour and paratumour tissues. A total of 28 397 epithelial cells were identified and classified into seven subclusters designated as E1–E7, and the subcluster distribution of sample pathology was visualised by the t‐SNE plot (Figure 2A). E6–E7 were enriched in tumour samples, while a higher fraction of E1, E2 and E3 was observed in paratumour samples (Figures 2B and C). GO terms were enriched in normal epithelial functions, such as epidemic development and cornification in E1, E2 and E3. E4 was enriched in ATP metabolism, and E5–E7 were enriched in RNA splicing (Figure 2D). Although there was no obvious difference in gene expression profiles across cell subclusters, a gradual increase in the expression level of oncogenes with a gradual decrease in tumour suppressors was identified. For example, TMPRSS11B, MUC21 and CRNN were highly expressed in the upper right corner of the t‐SNE plot characterised by E1, while the expressions of SOX4, MDK and IFI6 were higher in the upper left corner characterised by E6 and E7 (Figure 2D), which was similar to progressive ESCC.^21^ DSC2, RT4 and IL1RN were highly expressed in subclusters other than E6 or E7 (Figure S1B). Interestingly, when assessing the proportion of epithelial cells that contributed to each cluster by each patient sample, E7 was found not to be present in patient 3 (Figure 2F). Moreover, the pathological diagnosis of patient 3 was high‐grade intraepithelial neoplasia, while all the others were carcinoma. This finding further confirmed the hypothesis of E7 as a subcluster of malignant cells. In addition, the proportion of E7 was also positively correlated with an increase in the depth of tumour invasion (Table S1), rather than the size of microscopic lesion, indicating the potential relationship between E7 and tumour invasion. Notably, the suggested malignant E7 subcluster and potentially malignant E4, E5 and E6 subclusters also appeared in paratumour tissues (Figure S2C). These results suggested that the paratumour tissue could share an extensive overlap with intramucosal ESCC. CNV analysis of epithelial cells was performed and Bayes normalisation was applied (Figures 2G and S2A). E1 showed lower variation in CNV compared with other subclusters, indicating the benign nature of this subcluster (Figure S2A). Two major CNV patterns were observed in E2‐E7, one pattern with an obvious CNV gain in the anterior end of chromosome 1 and the other pattern with a significant CNV gain in the posterior end of chromosome 3 and an apparent CNV loss in chromosome 4 and chromosome 9. Notably, the second pattern was consistent with the previously reported genomic alterations in ESCC.^7^ Comparing CNV alterations in intramucosal ESCC with those in progressive ESCC, it was found that the second pattern in the current study was similar to the malignant cluster 3 in a recently published study, regardless that CNV was increased in chromosome 9 while decreased in malignant cluster 3 in progressive ESCC.^22^ Although the two CNV patterns both appeared in the E2–E7 subclusters, the proportion of the second pattern was higher in E7 subcluster at a single‐cell resolution. After Bayes normalisation, E7 exhibited a unique gain in CNV at the posterior end of chromosome 20 and the middle of chromosome 3. The CNV signal at the anterior end of chromosome 6 was highest in E7 and also increased in E5 and E6.

Afterwards, pseudotemporal and PCA analysis was performed to identify two evolutionary fates of oesophageal epithelial cells during ESCC tumorigenesis. As mentioned above, E4 is a proliferating cell population, and it could be inferred that the evolution initiated from proliferative E4, and then through an intermediate state characterised by E5, to two different terminal states of normal differentiated E1/E2/E3 or malignant E6/E7, respectively (Figure 2H). This was also similar to the previously reported cell fate of epithelial cell status transition in the mouse ESCC model.^9^ Gene expression in the sphingolipid metabolism pathway of E7 was shown along the trajectory (Figure 2I).

### 3.3 Metabolic characteristics of epithelial cells in human intramucosal ESCC

Aberrant metabolism is a major hallmark of cancer. Abnormal cancer metabolisms, such as aerobic glycolysis and increased anabolic pathways, play an important role in tumorigenesis, metastasis, drug resistance and cancer stem cells.^23^ To understand the metabolic landscape of epithelial cells in intramucosal ESCC, the scores of all 76 active metabolic pathways in E1–E7 clusters were first computed and 24 representing metabolic pathways were selected using the scMetabolism.^15^ There was a significant metabolic change trend from E1 to E7. E7 (a group of characteristic cells prone to malignancy) showed significantly different characteristics compared with other epithelial cell clusters.

**FIGURE 3:**
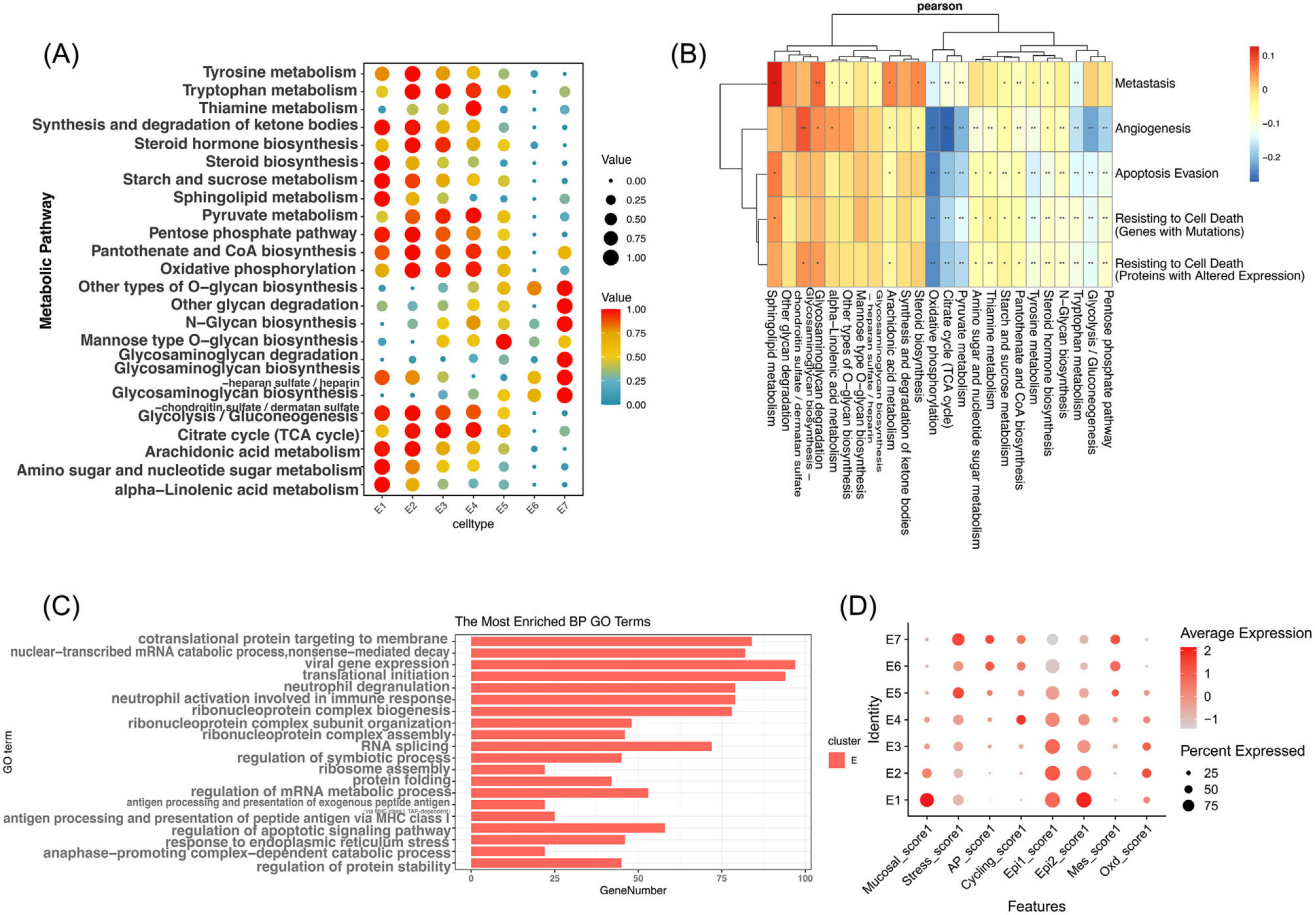
Characreristics of epithelial cells in human intramucosal ESCC. (A) Dotplot showing metabolic characteristics of seven subclusters, both colour and size indicate the effect size. (B) Correlation between the 24 representing metabolic pathways and phenotypic scores of E7 subcluster (^*^ indicates a *p* value < .05 and ^**^ < .01). (C) GO analysis for progressive ESCC compared with E1–E7 subclusters. (D) Dotplot showing similarities and differences between early and progressive ESCC. ESCC: oesophageal squamous cell carcinoma; GO: Gene Ontology.

The levels of glycolysis, pyruvate metabolism, oxidative phosphorylation, amino acid metabolism (e.g., tyrosine metabolism) and lipid metabolism (e.g., steroid biosynthesis) were lower in E7 than in other epithelial clusters (Figure 3A), which was different from progressive cancer.^22^ Similar to progressive ESCC, a significant increase in glycan biosynthesis and metabolism (e.g., glycosaminoglycan degradation/biosynthesis, glycan biosynthesis for other types of O) was observed in E7.

To explore the relationship between E7 and tumour progression, the correlation between metabolic pathways and tumour phenotypes was analysed by computing the classical phenotypic scores of cancer cells (Figure 3B). Through the correlation of phenotypic scores and metabolic pathways, markers of E7 were found to be strongly associated with metastasis and angiogenesis, which was not significant in other phenotypes such as cell proliferation.

Carbohydrate metabolisms and energy metabolisms, such as pyruvate metabolism, citrate cycle (TCA cycle) and oxidative phosphorylation in E7, showed a strong negative correlation with all phenotypes, which was opposite to the progressive ESCC.

Sphingolipid metabolism and glycan biosynthesis/metabolism (e.g., glycosaminoglycan degradation/biosynthesis) showed a strong positive correlation with metastasis and angiogenesis, which was consistent with previous studies.^24^ After searching for marker genes, it was further found that marker genes such as KDSR, DESG1, PLPP1 and NEU1 were related to sphingolipid metabolism in E7. KDSR and DESG1 are important molecules in the sphingosine synthesis pathway, which plays an important role in sphingolipid metabolism for the growth and metabolism of cancer cells.^24^ Gene expression in the sphingolipid metabolism pathway of E7 was shown along the trajectory (Figure 2I). The expression of KDSR was increased along the malignant trajectory, and NEU1 was significantly highlighted in E7 cells.

### 3.4 Unique characteristics of epithelial cells in human intramucosal ESCC compared with progressive ESCC

To understand the different characteristics between intramucosal and progressive ESCC, the data from both stages were combined to find the top markers of progressive ESCC^25^ (Figure S2E). Then, GO analysis was further performed on the top genes of malignant cells from progressive ESCC (Figure 3C), where biological processes related to protein synthesis and transport (e.g., ‘cotranslational protein targeting to membrane’, ‘translational initiation’ and ‘ribonucleoprotein complex biogenesis’), activated immune response (e.g., ‘neutrophil activation involved in immune response’, ‘neutrophil degranulation’ and ‘viral gene expression’) and RNA activities (e.g., ‘nuclear‐transcribed mRNA catabolic process’ and ‘RNA splicing’) were enriched. Although the GO terms related to RNA activities ranked relatively low in progressive oesophageal cancer, they were enriched during the malignant transition with high frequency, especially in potential malignant subclusters (E6 and E7). GO terms such as ‘cotranslational protein targeting to membrane’, ‘translational initiation’ and ‘ribonucleoprotein complex biogenesis’ were most frequently enriched in progressive ESCC, indicating the active protein synthesis in progressive cancer cells, which may be the next stage of malignant transition compared with RNA activities.

Additionally, the differences and similarities between intramucosal and progressive ESCC were also investigated by calculating the scores of epithelial subcluster functions (Figure 3D). In progressive ESCC, ‘MES’ was defined as related to the activation of epithelial–mesenchymal transition (EMT) and angiogenesis pathways.^25^ Interestingly, this subcluster was highly associated with E7 in intramucosal ESCC, which was analysed as strongly associated with metastasis and angiogenesis, although the proportion of ‘MES’ was substantially higher in progressive ESCC than that of E7 in early ESCC. The similarity between E7 and ‘MES’ suggested that highly malignant epithelial cells were also present in early ESCC.

Taken together, all the above results demonstrated that early ESCC shared both similarities and differences with progressive cancer and enhanced sphingolipid metabolism in E7 showed a strong potential for tumour metastasis and angiogenesis. Several molecules of the pathway were found to be potential early targets for the development of anti‐tumour strategies by further analysis.

### 3.5 Exhausted T cells and the suppressive immune microenvironment in human intramucosal ESCC

**FIGURE 4:**
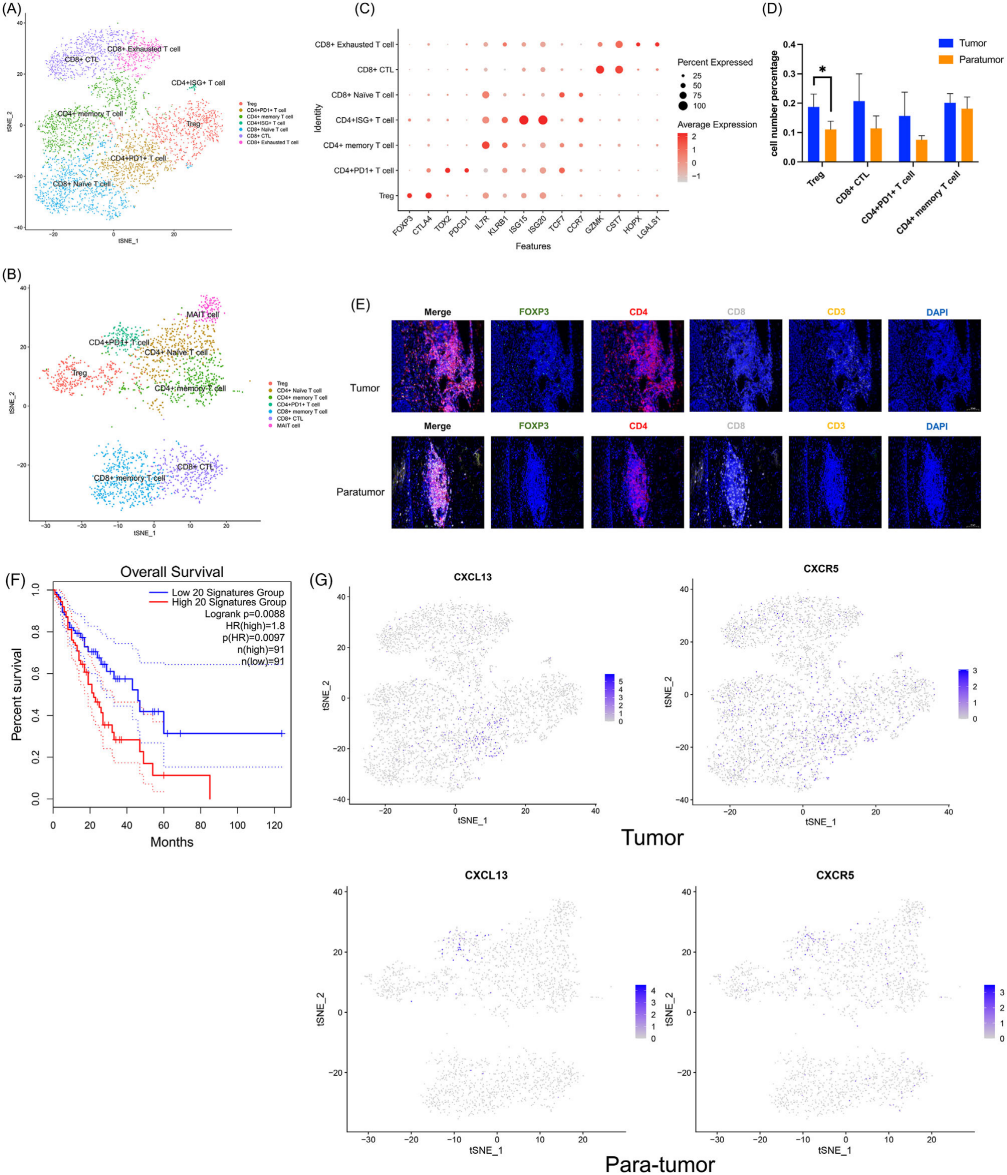
Exhausted T cells and suppressive immune microenvironment in human intramucosal ESCC. (A,B) t‐SNE plots revealing T cell subclusters in tumour specimens (A) and paratumour specimens (B). (C) Dotplot showing important markers of T cell subclusters, both colour and size indicate the effect size. (D) Distribution of T cell subclusters in different specimens (^*^ indicates a *p* value < .05). (E) Immunostaining for FOXP3, CD4, CD8 and CD3 showing the distribution of FOXP3+ cells in tumour and paratumour tissues of early ESCC patients. (F) Kaplan–Meier analysis showing overall survival of the top 20 PD1+CD4+ T cells markers in ESCC patients. (G) CXCL13 and CXCR5 expression in PD1+CD4+T cells shown by t‐SNE plots. ESCC: oesophageal squamous cell carcinoma; t‐SNE: t‐distributed stochastic neighbour embedding.

Due to the vast majority of T cells derived from PBMCs, T cells were clustered independently for tumour and paratumour tissues, to avoid the loss of signals from tissue samples (Figures 4A and B). Four T cell clusters appeared in both tumour and paratumour tissues (Figures 4A and B), including Tregs expressing FOXP3 and CTLA4,^26^ CD8^+^ CTLs expressing CST7, PD1^+^CD4^+^ T cells expressing PDCD1,^8^ and CD4^+^ T_M_s (memory T cells) (Figures 4C and S3B). Among them, there was a statistically significant difference in the number of suppressive T cells in tumour and paratumour tissues (Figure 4D), which was also verified by multiplexed immunohistochemical (mIHC) staining (Figure 4E). CD8+ T_EX_s expressing CST7 instead of CTLA4 or PDCD1 was detected in tumour tissues (Figures 4A and C). However, paratumour tissues did not form a separate CD8+ T_EX_s cluster due to the limited cell number (Figure S3C), which was also verified by mIHC (Figure 4E). T cells from tumour and paratumour tissues also exclusively exhibited distinct clusters. CD8^+^ T_M_s expressing CCL5 and GZMA, MAIT (mucosal‐associated invariant T) cells expressing TCF7 and PLAC8, and CD4^+^ T_N_s (Naïve T cells) expressing CCR7 appeared in paratumour tissues, while CD4^+^ISG^+^ T cells expressing ISG15/20 and CD8^+^ T_N_s expressing TCF7 only appeared in tumour tissues (Figures 4A, B, C and S3B, E). The proportion of T cell clusters in paratumour tissues from patient 3 was significantly different from other patients. He had a higher proportion of CD4^+^ T_M_s, PD1^+^CD4^+^ T cells, and MAIT cells, but a decreased proportion of CD8^+^ T_M_s (Figure S3F). Suppressive T cells in early ESCC demonstrated unique metabolic characteristics. Genetically, enhancement in glycolysis/gluconeogenesis and oxidative phosphorylation was observed in PD1+ CD4+ T cells. However, exhausted CD8 T cells had a significantly lower metabolic level (Supplementary Results and Figures S3G–I).

More importantly, PD1^+^CD4^+^ T cells were found to be associated with more numbers in tumour tissues than paratumour tissues, and the top 20 markers of these cells in tumour tissues (logfc > 0.95, *p* < .001) were associated with a lower probability of overall survival in ESCC (Figure 4F). However, there was no significant correlation between the top markers of PD1^+^CD4^+^ T cells in paratumour tissues and the survival of oesophageal cancer. Furthermore, the specific high expression of CXCL13 and CXCR5 in PD1^+^CD4^+^ T cells was observed in tumour tissues as well as adjacent tissues (Figure 4G).

Taken together, the abundance of CD8^+^ T_EX_s and the enrichment of Tregs and PD1^+^CD4^+^ T cells suggested exhausted and suppressive immune microenvironment in human intramucosal ESCC.

### 3.6 Characterisation of myeloid cell differentiation

**FIGURE 5:**
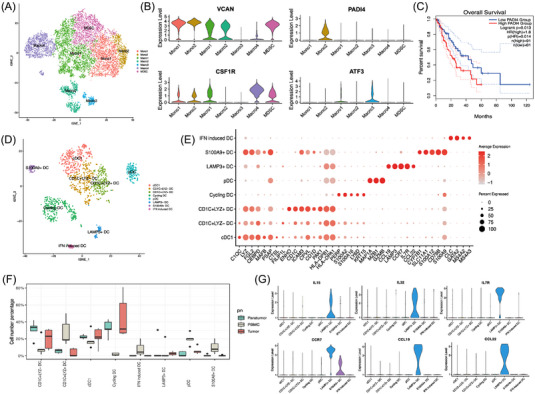
Characterisation of myeloid cell subpopulations. (A) t‐SNE plot showing seven monocyte/macrophage cell subclusters. (B) Violin plots showing gene signatures of MM cell subclusters. (C) Kaplan–Meier analysis showing overall survival of PADI4 in ESCC patients. (D) t‐SNE plot showing dentric cell subclusters. (E) heatmap showing important gene signatures of DC subclusters. (F) Distribution of DC subclusters in different specimens. (G) Violin plots showing immune‐relevant genes expressed by LAMP3+ DCs. (F) Cell number percentage in different specimens. ESCC: oesophageal squamous cell carcinoma; t‐SNE: t‐distributed stochastic neighbour embedding; MM: monocyte/macrophage; DC: dendritic cells.

Seven monocyte/macrophage cell clusters were categorised (Figure 5A). Both Mono1 and Mono2 showed a strong monocyte signature. Mono1 had a high expression of VCAN, while Mono2 had a high expression of PADI4 (Figure 5B). Interestingly, PADI4 was strongly associated with a high probability of overall survival in ESCC (Figure 5C), suggesting it is a prognostic biomarker in ESCC. Human PADI4 was first described in HL‐60 cells and was found to participate in the differentiation of HL‐60 cells into granulocytes and monocytes/macrophages.^27^

Eight DC clusters were characterised according to discriminative markers, including cDC1, CD1C^+^LYZ^−^ DC, CD1C^+^LYZ^+^ DC, pDC, cycling DC, LAMP3^+^ DC, S100A9^+^ DC and IFN‐induced DC (Figures 5D and S4C). cDC1 was characterised by a high expression of PSAP, FGL2 and HLA‐DPA1,^28^ whereas pDC was enriched with GZMB, MAP1A and MZB1^29^ (Figure 5E). The two subclusters were consistent with the expression profile of the original classification of conventional DC1 and pDC. Both CD1C^+^LYZ^−^ DC and CD1C^+^LYZ^+^ DC had a high expression of CD1C, which were considered members of cDC2. Moreover, CD1C^+^ DCs were further distributed into two clusters with a similar number of cells. Compared with CD1C^+^LYZ^−^ DC, CD1C^+^LYZ^+^ DC had a strong signature of acute and chronic inflammatory genes, such as LYZ, ICAM3 and HLA‐DPB1 (Figure 5E). Interestingly, the proportion of CD1C^+^LYZ^+^ DC was lower in tissues compared with PBMCs, while the proportion of CD1C^+^LYZ^−^ DC was higher in tissues (Figure 5F). CD1C^+^LYZ^−^ DC and CD1C^+^LYZ^+^ DC subclusters were similar to CD1C_A and CD1C_B subclusters defined by Villani et al.^28^ S100A9^+^ DC showed a high expression of S100A9, CYP1B1, S100A12, etc. IFN induced DC was characterised by a high expression of CLC, GATA2, MS4A2 and MS4A3, which was similar to an IFN induced DC subset in muring DC system.^30^ Based on the GO analysis, another subcluster was named cycling DC (Figure S1C), suggesting that this subcluster was in the cell cycle. This subgroup was also enriched in tissues compared with PBMCs (Figure 5F). LAMP3^+^ DC was recently recognised by several studies^31^, ^32^, ^33^ as a tumour‐specific DC subcluster, expressing a variety of immune‐relevant genes encoding cytokines (IL7R, IL15, IL32), chemokines and chemokine receptors (CCR7, CCL19, CCL22) (Figures 5E and G).^34^

### 3.7 Distinct fibroblast subclusters in human intramucosal ESCC ecosystem

**FIGURE 6:**
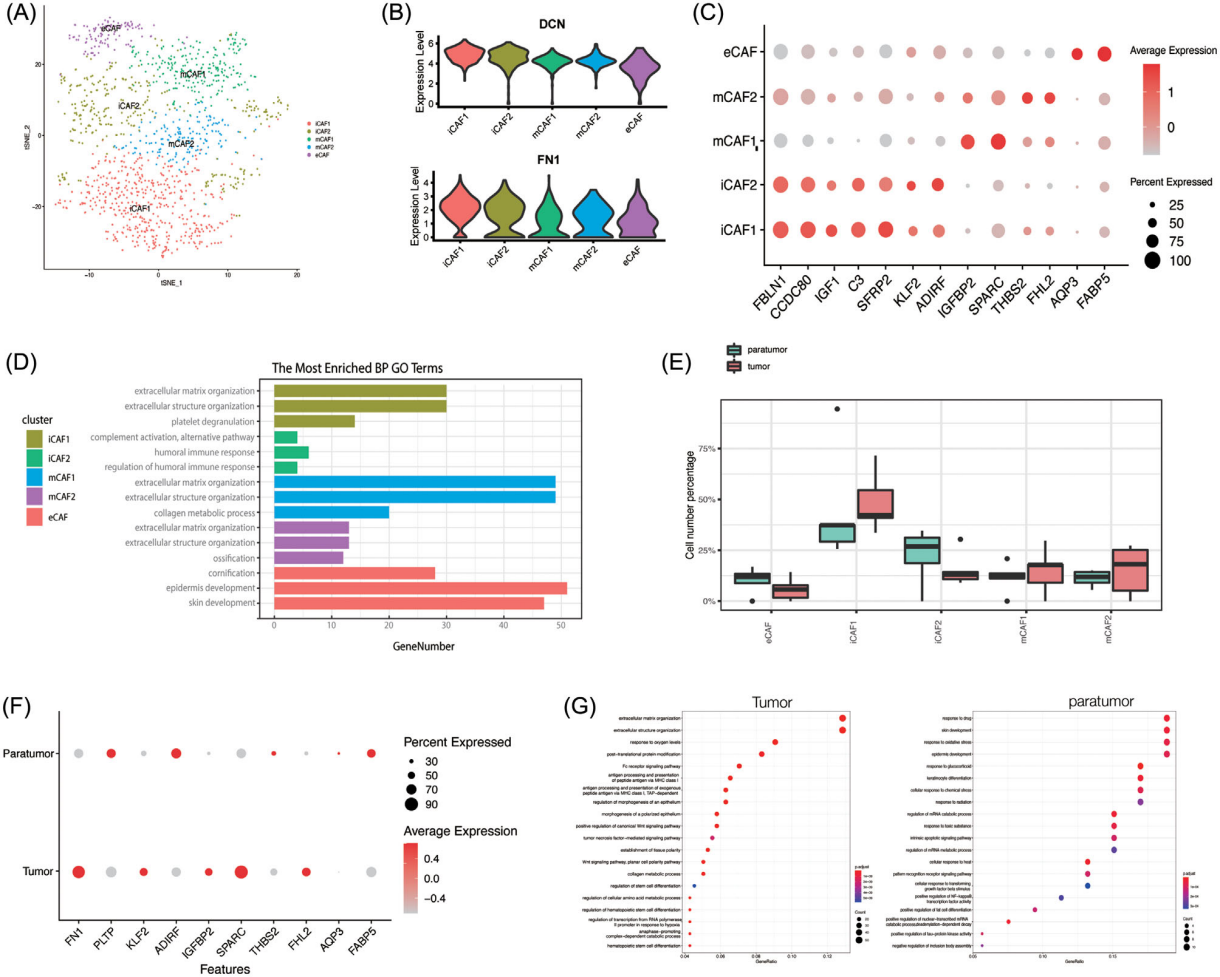
Distinct fibroblast subpopulations in human intramucosal ESCC ecosystem. (A) t‐SNE plotshowing five subclusters of cancer‐associated fibroblasts. (B) Violin plot showing important gene signatures expressed by all the five CAF subclusters collectively. (C) Dotplot showing important gene signatures of CAF subclusters respectively, both colour and size indicate the effect size. (D) GO analysis for CAF subclusters. (E) Distribution of CAF subcusters in different specimens. (F) Dotplot showing important gene signatures of CAFs derived from tumour and paratumour specimens. (G) GO analysis for CAF derived from tumour and paratumour specimens. ESCC: oesophageal squamous cell carcinoma; t‐SNE: t‐distributed stochastic neighbour embedding; CAF: cancer‐associated fibroblast; GO: Gene Ontology.

A total of 1279 CAFs were clustered into five subclusters (Figure 6A). All five subclusters expressed high levels of fibroblast markers, such as ACTA2 (a‐SMA), PDGFRa, DCN, FN1 and LUM (Figure 6B), while each subcluster displayed distinct transcriptomic signatures. iCAF1 and iCAF2 expressed high levels of FBLN1, CCDC80, IGFI, IGFBP6, CFD, SFRP2 and complement genes (C3 and C7) (Figures 6C and S4D). Accordingly, fibroblasts in the two subclusters were named inflammatory CAFs.^35^ Interestingly, although the GO terms enriched for iCAF1 and iCAF2 had common pathways related to extracellular matrix tissue and extracellular structure tissue (Figure 6D), the pathways related to inflammatory response were opposite. Specifically, iCAF1 participated in the negative regulation of the immune system process, while iCAF2 participated in complement activation and complement‐dependent cytotoxicity. Therefore, the role of iCAF1 was further identified as inhibition and iCAF2 as activation in immune modulation. Moreover, iCAF1 was mainly enriched in ESCC tissues, whereas the proportion of iCAF2 was relatively higher in paratumour tissues, which was also consistent with the status of suppressed immune in tumorgenesis (Figure 6E). The active metabolism of iCAF1 had a high level of lipid metabolism through scMetabolism (Figure S5), including glycosphingolipid biosynthesis and glycerophospholipid metabolism. It was proposed that the enhancement of lipid metabolism of CAFs promoted tumour metastasis.^36^ mCAF1 and mCAF2 expressed high levels of extracellular matrix signatures, including POSTN, MMP11, MMP14, and collagen molecules, including COL3A1, COL1A1, COL5A1, together with GO terms enriched with extracellular matrix tissue, extracellular structure tissue, ossification and collagen fibril tissue (Figure 6D). Therefore, they were designated as matrix CAFs.^37^ eCAF mainly expressed epithelium‐specific marker genes, such as KRT15, KRT 6B, KRT6A and KRT19, which was consistent with EMT‐like CAFs in a previous report.^38^ GO analysis of this subcluster also revealed enrichment in epidermis and skin development, as well as energy‐related pathways such as ATP metabolic process and mitochondrial transport (Figure 6D). Thus, this subgroup was named eCAF to account for its relationship with energy and EMT. ScMetabolism also displayed a significant increase in the strong metabolic activity of eCAF (Figure S5). Therefore, it can be speculated that this cell cluster may be associated with the proliferation and biosynthesis of tumour‐related fibroblasts and may communicate with tumours. CAFs derived from tumour specimens demonstrated different gene signatures from those derived from paratumour specimens (Figure 6F), and GO analysis also showed different pathway enrichment (Figure 6G). CAFs derived from tumour specimens were associated with higher signals in extracellular matrix tissue, extracellular structure tissue, antigen processing and response to oxygen levels. Previous reports have also highlighted a subcluster called vCAF in malignant tumours,^37^, ^38^ characterised by microvasculature signature genes such as CD146 (MCAM), MYH11 and RGS5, as well as inflammatory chemokines such as IL‐6 and CCL8, with functions mainly in response to hypoxia and mesenchymal cell proliferation. However, this subcluster was not found in human intramucosal ESCC, which may be related to the biological behaviour at the very early stage of ESCC.

### 3.8 Interaction of E7 cells and CAFs via the MDK–NCL pathway

To explore the interaction between T cells, epithelial cells and CAFs, intercellular interaction analysis was conducted based on ligand–receptor pairs (Figures 7A–C). Different from progressive ESCC, the interaction between different cell types decreased significantly due to the early stage of tumour.^22^ In the network of intramucosal ESCC, the interaction between epithelial cells and CAFs was predicted to be the most significant. An increased intercellular interaction in MDK (midkine)–SDC2 (syndecan‐2), MDK–NCL (nucleolin) and MDK–LRP1 (LDL receptor related protein 1) axes was observed between epithelial cells and CAFs, and the interaction between E7 cluster and eCAFs/iCAFs was observed to be the most prominent. In the MDK–NCL axis, E7 showed a particularly strong intercellular interaction with eCAF and mCAF2, however, with iCAF1 and iCAF2 in the MDK–LRP1 axis (Figure 7D).

FIGURE 7Altered crosstalk between subclusters in human intramucosal ESCC. (A) Circos plots showing crosstalk between T cell and CAF subclusters. (B) Circos plots showing crosstalk between epithelial cells and CAF subclusters. (C) Circos plots showing crosstalk between T cell and epithelial cell subclusters. (D) Dotplot showing upregulated and downregulated interactions between epithelial cells and CAF subclusters. (E) Expression level of MDK, NCL and LRP1 in human oesophageal cancer and paratumour specimen, data from GEPIA. (F) Correlation analyses between MDK and NCL, and between MDK and LRP1, data from GEPIA. (G) Schematic illustration of intercellular crosstalk between early ESCC cells and CAFs. (H) CCK‐8 assays were conducted to determine the function of MDK–NCL on proliferation capabilities. Proliferation of CAFs was significantly promoted by the stimulation of tumour supernatant and the alteration could be rescued by iMDK. (I) Knockdown on NCL protein levels in d042 cell lines transfected with NC and siNCL. (J) CCK‐8 assays were conducted to determine the function of MDK–NCL on proliferation capabilities. Knockdown of NCL reversed the tumour supernatants stimulation to CAFs proliferation. (K) HE staining and IHC in ESCC specimen of paratumour specimen, high grade intraepithelial neoplasia, intramucosal ESCC and progressive ESCC. All data are presented as mean ± SD and all the experiments were repeated three times. Data were analysed by unpaired *t*‐test. ^*^ *p* < .05, ^**^ *p* < .01, ^***^ *p* < .001. ESCC: oesophageal squamous cell carcinoma; CAF: cancer‐associated fibroblast; GO: Gene Ontology; GEPIA: Gene Expression Profiling Interactive Analysis; HE: hematoxylin–eosin; IHC: immunohistochemistry.

**Figure:**
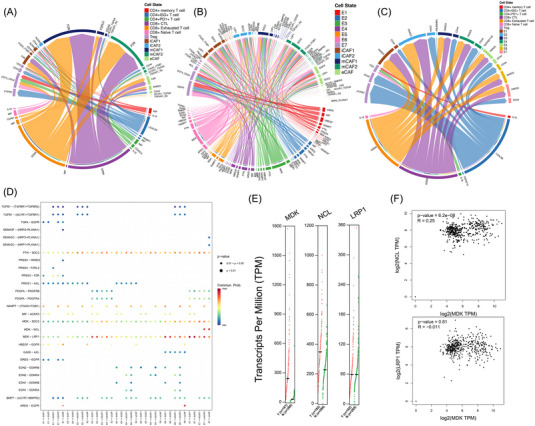

**Figure:**
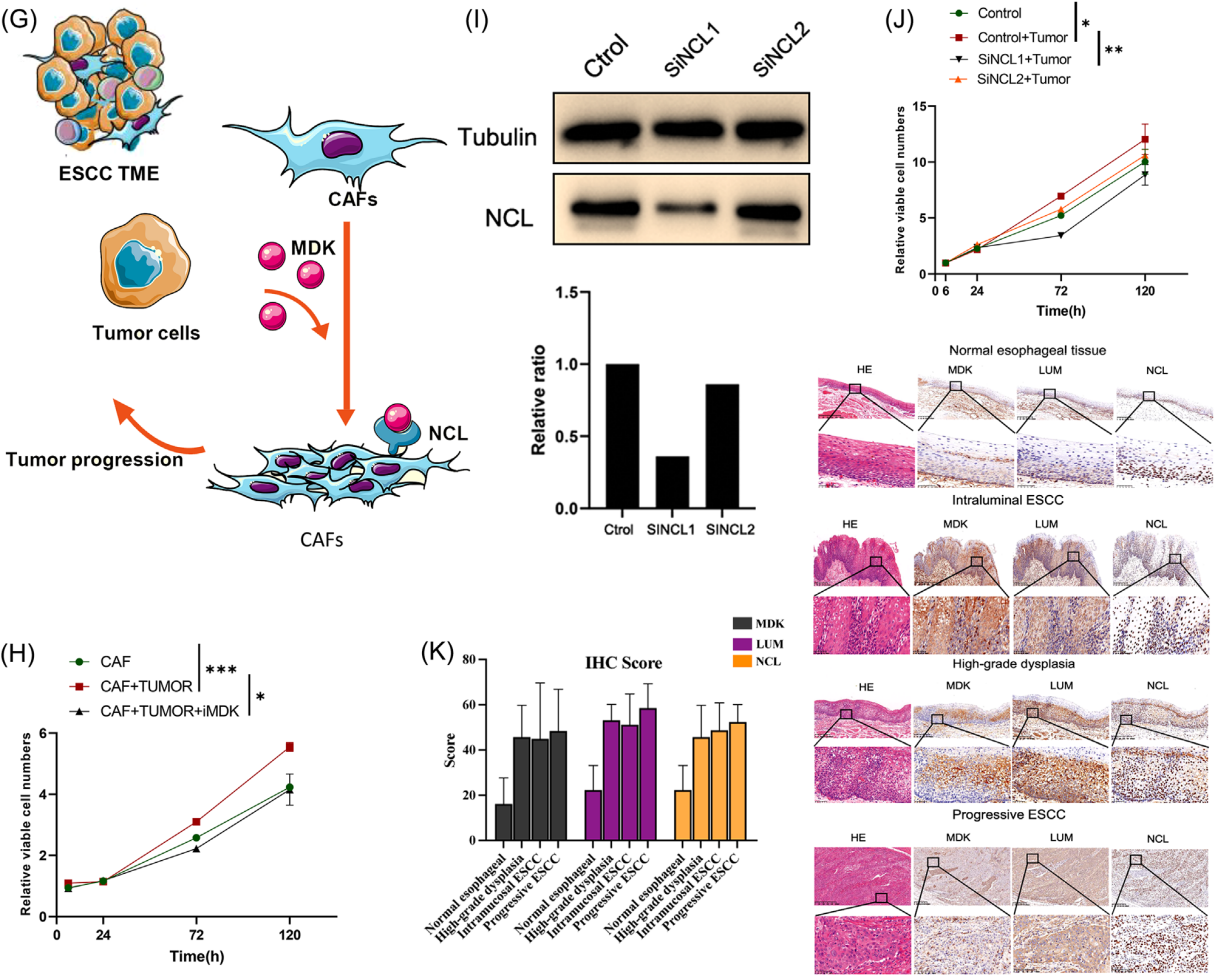

The expression levels of MDK, NCL and LRP1 were then determined using the TCGA database. The results suggested the expression level of MDK in human oesophageal cancer was more than eight times higher than that in paratumour tissue, and the expression of NCL was also slightly increased in human oesophageal cancer. However, there was no significant difference for LRP1 (Figure 7E). Through correlation analysis using data from TCGA and GTEx, the expression level of MDK was significantly correlated with NCL in human oesophageal cancer and paratumour oesophageal tissues. Meanwhile, the expression level of MDK was not significantly correlated with LRP1 (Figure 7F). Therefore, the hypothesis that MDK in oesophageal cancer cells could interact with NCL on the surface of CAFs to upregulate the proliferation of CAFs was proposed (Figure 7G). To investigate whether tumour cells promote the proliferation of CAFs by secreting MDK, CAFs were cultured with and without tumour cell supernatants, and the inhibitor of MDK (iMDK) was added to verify the role of MDK. The proliferation of CAFs was significantly promoted by the stimulation of tumour supernatants and the alteration could be rescued by iMDK (Figure 7H). Furthermore, NCL in CAFs was knocked down and cultured with tumour supernatants (Figure 7I). Expectedly, the knockdown of NCL reversed the stimulation of tumour supernatants to the proliferation of CAFs (Figure 7J). Then, IHC was further performed in the ESCC specimen of paratumour tissue, high‐grade dysplasia, intramucosal ESCC and progressive ESCC (Figure 7K and Table S2). Expectedly, with the progression of ESCC, the expression of MDK and NCL in tumour tissue increased step by step, and the close location indicated the interaction between CAFs and tumour.

Collectively, it can be seen from these results that MDK was upregulated in oesophageal epithelial cells during the malignancy change and interacted with NCL in mCAF and eCAF to promote the proliferation of CAFs. The enriched MDK–NCL signal could serve as a sign of CAF activation to stimulate downstream pathways for facilitating tumour invasion, and thus may be a potential early biomarker of ESCC progression.

For other cell types, potential strong interactions between CAFs and T cells were observed via ligand–receptor interaction, such as GZMA‐F2R (Figure 7A), to induce tumour suppression and T cell‐mediated killing of tumour cells between cytoxic T and tumour cells in hepatocellular carcinoma.^39^ In the present study, cluster mCAF1/2 received immune signals from suppressive T cells (CD8^+^ exthausted T cells and Treg) through GZMA and its receptor F2R instead, as a potential mediator of tumour microenvironment.

## 4 DISCUSSION

In this study, the single‐cell transcriptome and metabolism landscape of human intramucosal ESCC was systematically described. Paired samples of endoscopically resected intramucosal ESCC, para‐ESCC oesophageal tissue from endoscopically resected specimens, and PBMCs were adopted for scRNA‐seq. A recently developed computational pipeline, scMetabolism, was also applied to visualise and quantify the metabolic diversity of single cells. Due to the nonspecific symptoms of early ESCC, the detection of early ESCC almost completely depended on timely gastroscopy. Post ESD pathological diagnosis is crucial for the confirmation of treatment adequacy and further intervention or follow‐up. The minimal influence on pathological assessment was proposed as a prerequisite, with a limited sample volume from ESD specimens, and therefore scRNA‐seq was proposed as one of the most appropriate methods for describing early ESCC microenvironment. However, there is currently no transcriptome landscape description of human early ESCC at a single‐cell resolution.

The lower proportion of B cells in ESCC compared with other tumours was proposed by a previous study on progressive ESCC.^8^ Interestingly, the average proportion of infiltrating B cells in intramucosal ESCC was significantly higher than that in progressive ESCC. It further increased in patients of the earlier stage, which was consistent with previous studies,^20^ indicating the positive effects of tumour‐infiltrating B cells.

The characteristics of intramucosal ESCC share similarities and differences with progressive ESCC. Firstly, GO terms related to RNA and protein activities were enriched in both intramucosal and progressive ESCC, and RNA splicing ranked high in E6 and E7. However, GO terms related to protein synthesis and transport were most frequently enriched in progressive ESCC, indicating the active protein synthesis in progressive cancer cells, which may be the next stage of malignant transition compared with RNA activities. Secondly, the scores of epithelial subcluster functions were calculated,^25^ finding that ‘MES’ related to the activation of EMT angiogenesis pathways in progressive ESCC shared great similarities with E7 in intramucosal ESCC, although the proportion was substantially higher in progressive ESCC than in early ESCC. Thirdly, exhausted T cells were observed in both intramucosal and progressive ESCC, but inconsistent exhaustion of CD4+ and CD8+ T cells occurred in intramucosal ESCC. The CD8+ TEXs expressed a high level of GZMK rather than typical exhaustion markers like PDCD1 or CTLA4, suggesting that CD8+ TEXs in intramucosal ESCC were mainly in an intermediate state before total exhaustion, and could become terminal TEX through the ‘Tn to IL7R+Tm to GZMK+TEM/EX’ pathway.^40^ Finally, metabolism also varied in both intramucosal and progressive ESCC. The levels of glycolysis, pyruvate metabolism, oxidative phosphorylation, amino acid metabolism and lipid metabolism remained low in E7,^25^ which was different from progressive cancer,^22^, ^25^, ^41^ indicating that intramucosal ESCC may not initiate a large‐scale cell growth and proliferation or suffer from nutrient and oxygen deprivation. It was also reported that limited oxidative phosphorylation is related to apoptosis and evasion,^41^ which was verified by the following correlation analysis. An increase in glycan biosynthesis and metabolism was observed in both progressive ESCC and E7. Glycans play a key role in cancer progression and treatment as glycosylation leads to various functional changes of glycoproteins that confer unique characteristic phenotypes associated with cancer cells,^42^ which was also verified by correlation analysis. Notably, the proportion of E7 was also positively correlated with an increase in the depth of tumour invasion, which supported further investigation of the relationship between metabolic changes and invasive phenotypes. Correlation analysis suggested that sphingolipid metabolism showed a strong correlation with cancer metastasis, which was consistent with previous studies.^6^ Sphingosine synthesis in sphingolipid metabolism plays an important role in the growth and metabolism of cancer cells.^24^ Additionally, KDSR and NEU1 in this pathway was found to be significantly upregulated along the trajectory indicating potential targets for the development of anti‐tumour strategies.

The results suggested high inter‐tumour heterogeneity and epithelial cells in human intramucosal ESCC. The subclusters of epithelial cells demonstrated a transitional cell status, in which only a small portion of cells were malignant tumour cells, while the vast majority were still in the way of malignancy changes. Previous research focused on high‐grade intraepithelial neoplasia and proposed that HIN largely retains the transcriptional and functional features of the normal epithelium,^43^ which is in line with the results of this study. Meanwhile, paratumour tissues were not normal oesophageal tissues, without obvious boundary with neoplasia. Considering the sampling technique for choosing unstained areas in the ESD resected specimen, pre‐cancerous status like low‐grade intraepithelial neoplasia could also exist in paratumour tissues. Actually, in clinical work, lightly stained mucosa or suspicious satellite lesions often appeared surrounding the major unstained lesion, with pathological confirmation as the target for ESD. Our results revealed the abundance of potentially malignant cells in the paratumour tissue of ESCC, which may also help to explain the high risk of metachronous de novo carcinogenesis. This result is also consistent with previous findings that genomic mutations leading to the development of ESCC may also occur in normal pathologically paratumour human oesophageal tissues. More importantly, tumour‐adjacent paratumour epithelia shared an extensive overlap with dysphagia.^9^

To exclude the impact of abundant immune cells in PBMCs, T cells derived from tumour tissue, paratumour tissue and PBMCs were analysed separately, and a unique suppressive immune microenvironment was found in early ESCC. For monocytes and dendritic cells (Figure S4B), mixed data were demonstrated as when focusing on cells from tumour and paratumour tissues without PBMCs in the supplementary figure, the subclusters and gene signatures of each subcluster turned out to be consistent with the mixed data.

Intercellular interaction analysis based on ligand–receptor pairs revealed that malignant subclusters related to metastasis and angiogenesis interacted with CAFs via the MDK–NCL pathway. MDK is a member of a small family of secreted growth factors that binds heparin and responds to retinoic acid. It is currently considered a multifaceted factor contributing to both paratumour tissue homeostasis and disease development. MDK is highly expressed in various human malignancies and acts as a mediator for the acquisition of key cancer hallmarks, including cell growth, survival, metastasis, migration and angiogenesis.^44^ NCL is a multifunctional protein that is mainly localised in the nucleolus and is also found in the nucleoplasm, cytoplasm and cell membrane. NCL is found to interact with different proteins and RNA sequences for participation in several pathological processes, especially in tumorigenesis and EMT, which serves as a potential target for the development of anti‐tumour strategies.^45^ Regarding its function in fibroblasts, it was previously reported to regulate the phosphorylation and nuclear export of fibroblast growth factor 1,^46^ which acts as a paracrine regulator of oncogenic transcription.^47^ Previous literature on the interaction between MDK and NCL mainly focused on immunosuppression. MDK was also found to promote articular chondrocyte proliferation via the MDK–LRP1–NCL signaling pathway.^48^ Therefore, it was hypothesised that MDK in oesophageal cancer cells could interact with NCL on the surface of CAFs to upregulate the proliferation of CAFs. CAFs are key players in stromal cells for most solid tumours owing to the characteristic of abundance as well as enhanced proliferative and paracrine potential. In addition, the CAF‐mediated control of tumour progression includes mesenchymal‐dependent and mesenchymal‐independent mechanisms. The former relies on the induction of EMT in cancer cells through bidirectional crosstalk of paracrine signals (with specific engagement in deregulated pathways such as IL‐6, IL‐11 and TNF‐α) to activate stemness‐related pathways and metabolic reprogramming of tumour cells, while the latter promotes cancer cells for invasion of ECM by co‐migration or ECM remodeling to favour its metastatic potential.^49^ Notably, the enriched MDK–NCL signal was observed in two specific subclusters of CAFs. It was also found that mCAF1 was related to ECM organisation and eCAF was associated with energy metabolism and EMT. Although the abundance of these two subclusters was not increased in human intramucosal ESCC compared with paratumour tissue, the highly upregulated MDK–NCL pathway interacting with E7 cells was negligible compared with interactions with other epithelial cell clusters. As described above, E7 was a confirmed subcluster of malignant epithelial cells which was not found in high‐grade intraepithelial neoplasia. Moreover, MDK was highly expressed in the E7 subcluster. Cell proliferation assay demonstrated significant differences between CAFs with or without tumour supernatant/knockdown of NCL/MDK inhibitor, underlining the importance of MDK–NCL pathway in early ESCC microenvironment. IHC further confirmed the gradual increase of MDK and NCL in high‐grade dysplasia, intramucosal ESCC and progressive ESCC. Therefore, the activated MDK–NCL signal is an important hallmark in the change of tumour microenvironment when invasion and metastasis occur shortly with the help of proliferative CAFs. The enriched MDK–NCL signal could serve as a sign of CAF activation to stimulate downstream pathways for promoting tumour invasion, and therefore may be a potential early biomarker of ESCC progression.

There are also limitations to this study, which should be taken into consideration when interpreting the results. First, the limited sample size may lead to bias in the results. Second, the scarcity of early ESCC samples may bring difficulty in carrying out validation experiments on some specific clusters. Nevertheless, through the above efforts, it is believed that this study will contribute to the comprehension of TME and cellular heterogeneity in both early and progressive ESCC patients and will serve as a valuable resource to deeply explore the pathogenesis of ESCC and identify potential therapeutic targets for early ESCC in the future.

In conclusion, the cellular atlas of human intramucosal ESCC was proposed to pinpoint changes in cell subsets and transcriptional levels related to the initiation and development of early ESCC in the tumour microenvironment. The results of scRNA‐seq decipher a range of genes and signaling pathways with potential clinical application value for novel interventions, such as therapies targeting the MDK–NCL pathway. Therefore, further research into these genes will certainly bring new progress to the molecular diagnosis and immunotherapy of early ESCC.

## CONFLICT OF INTEREST STATEMENT

The authors declare no conflict of interest.

## Supporting information

Supplementary Information

Supplementary Information

Supplementary Information

Supplementary Information

Supplementary Information
