# Supplementary figures and images for: Single‐cell transcriptomic analysis deciphers key transitional signatures associated with oncogenic evolution in human intramucosal oesophageal squamous cell carcinoma
Source: Clin Transl Med. 2023 Feb 28;13(3):e1203. doi: 10.1002/ctm2.1203 (PMC9975454; doi:10.1002/ctm2.1203)

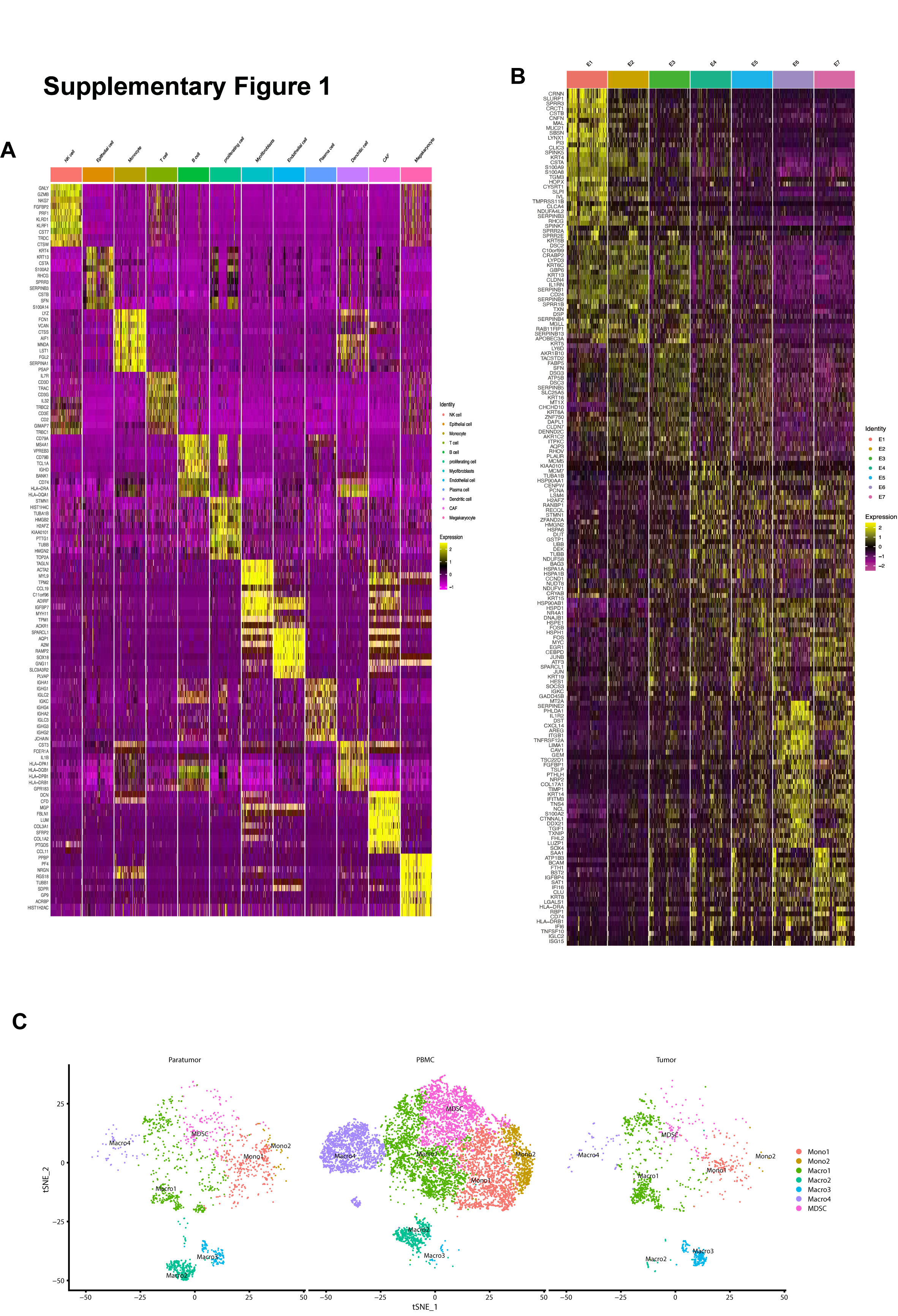

Supplement: Supplementary file 1 — Supplementary Information [file CTM2-13-e1203-s001.tif]

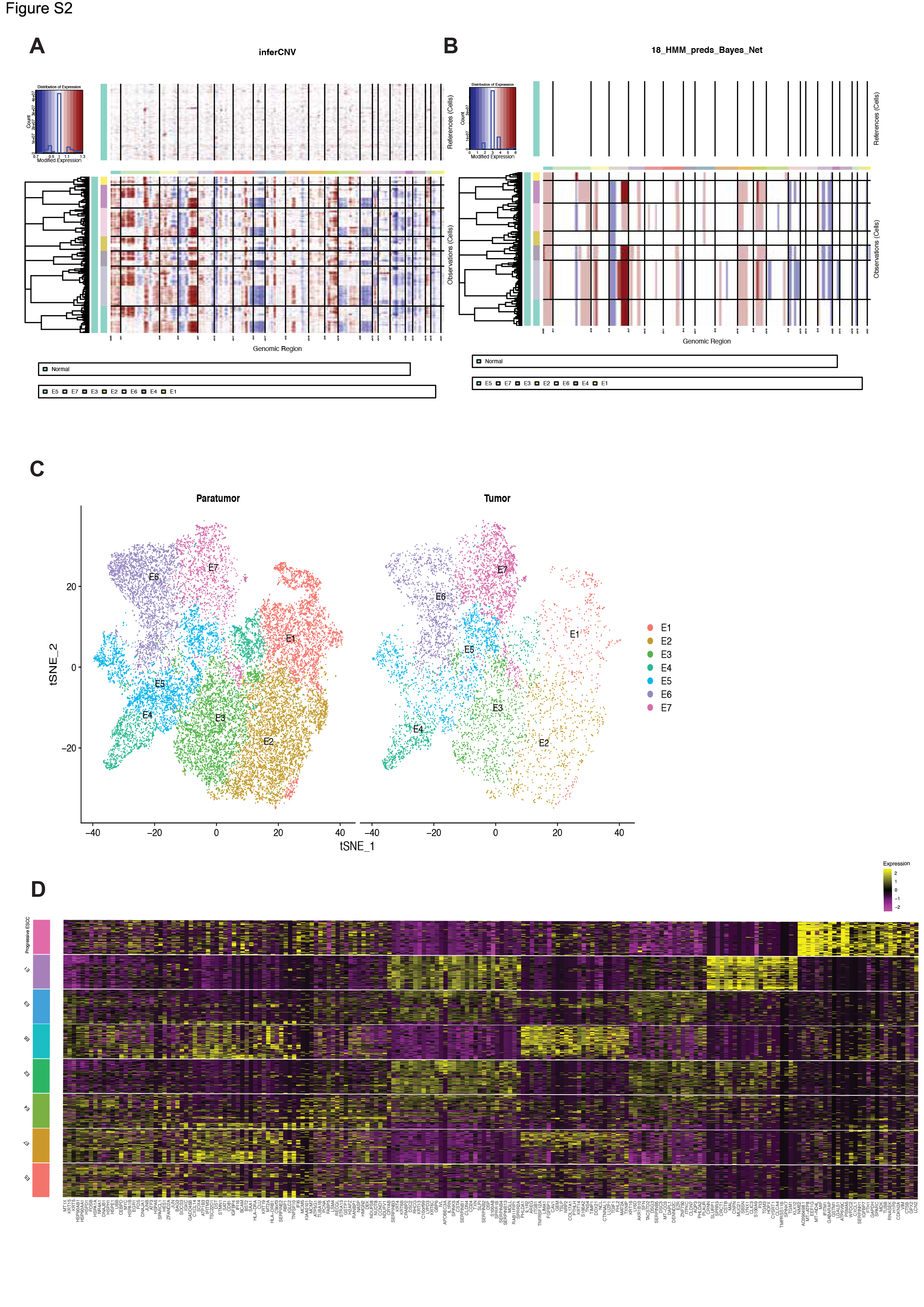

Supplement: Supplementary file 2 — Supplementary Information [file CTM2-13-e1203-s005.tif]

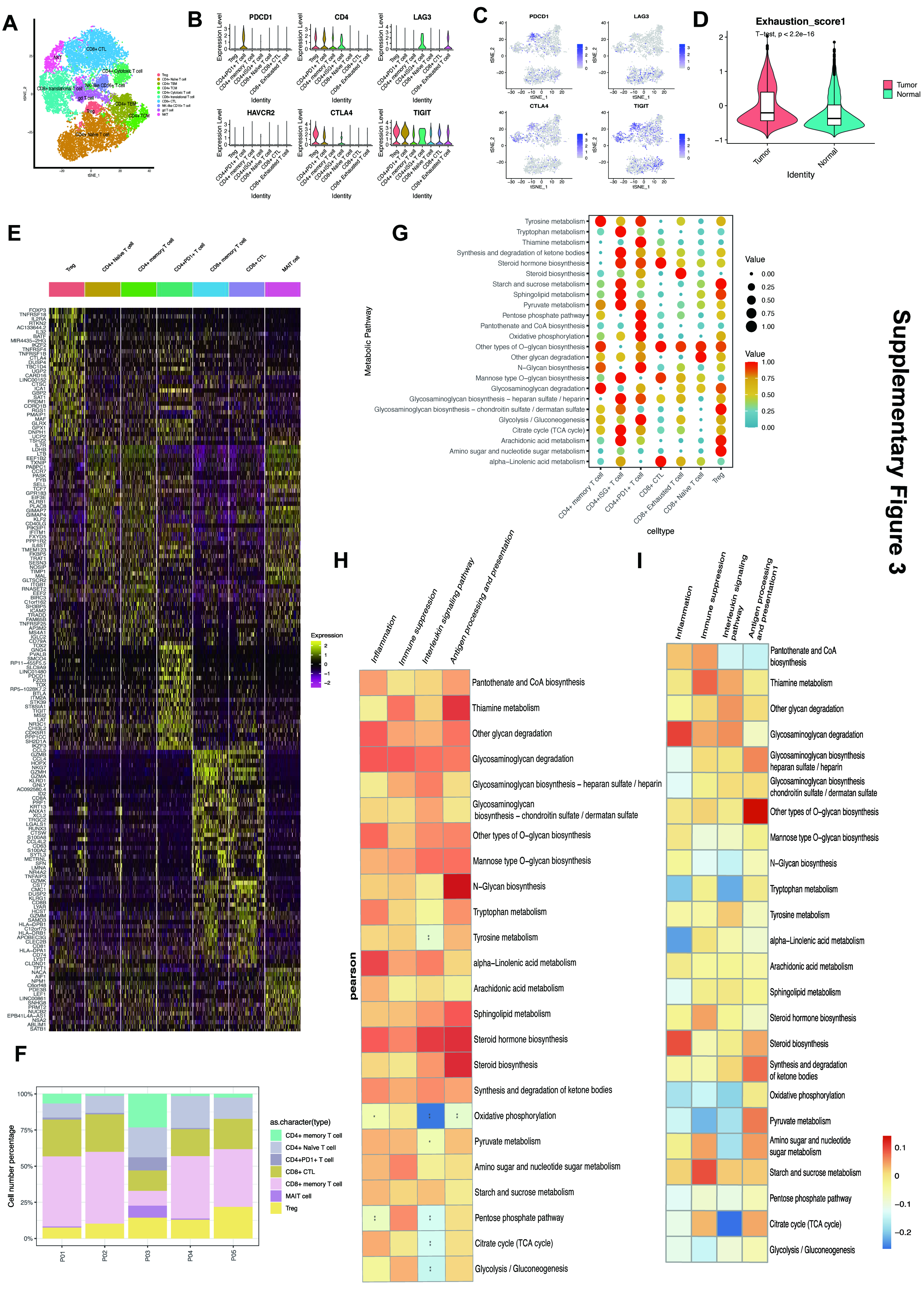

Supplement: Supplementary file 3 — Supplementary Information [file CTM2-13-e1203-s003.tif]

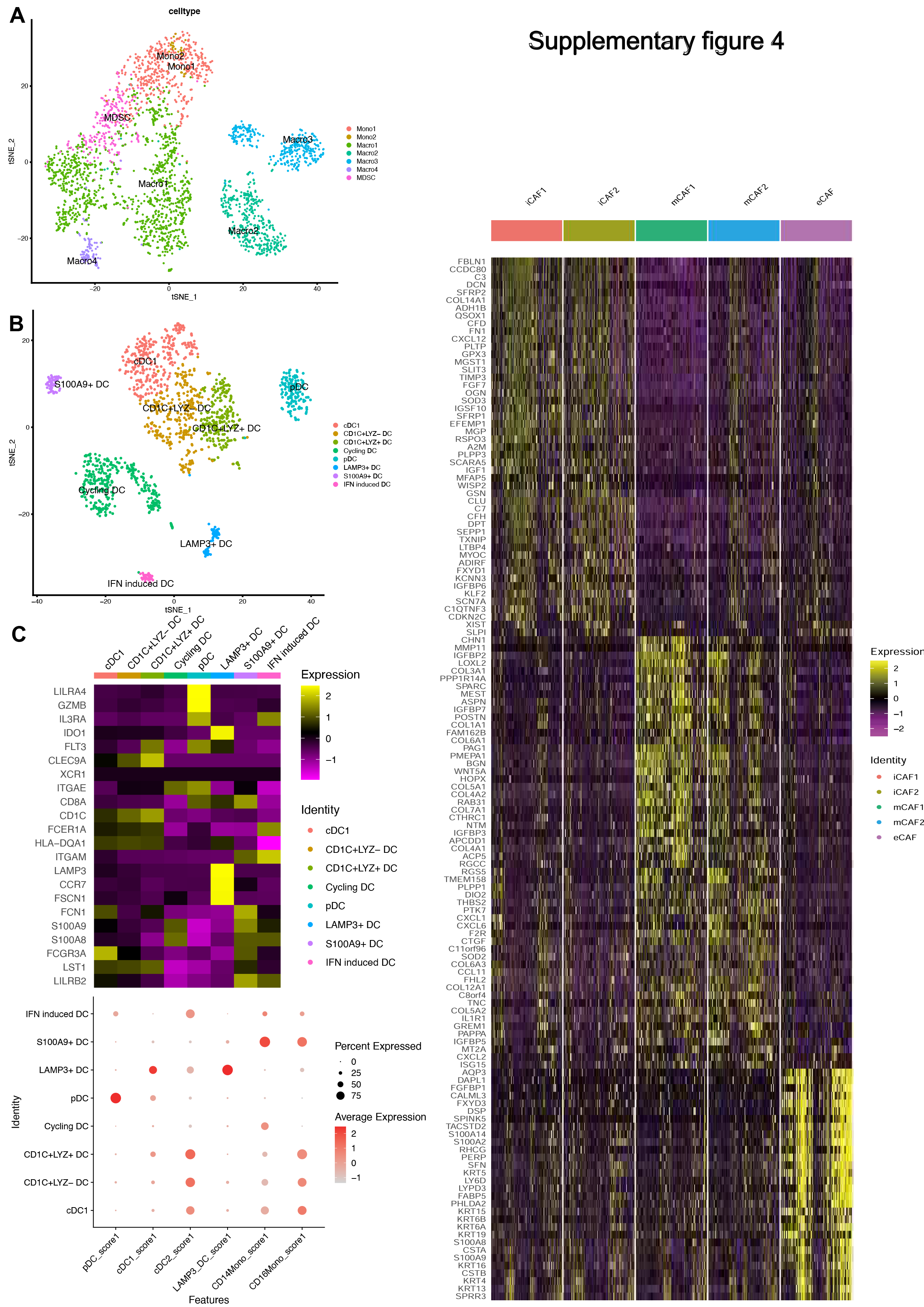

Supplement: Supplementary file 4 — Supplementary Information [file CTM2-13-e1203-s004.tif]

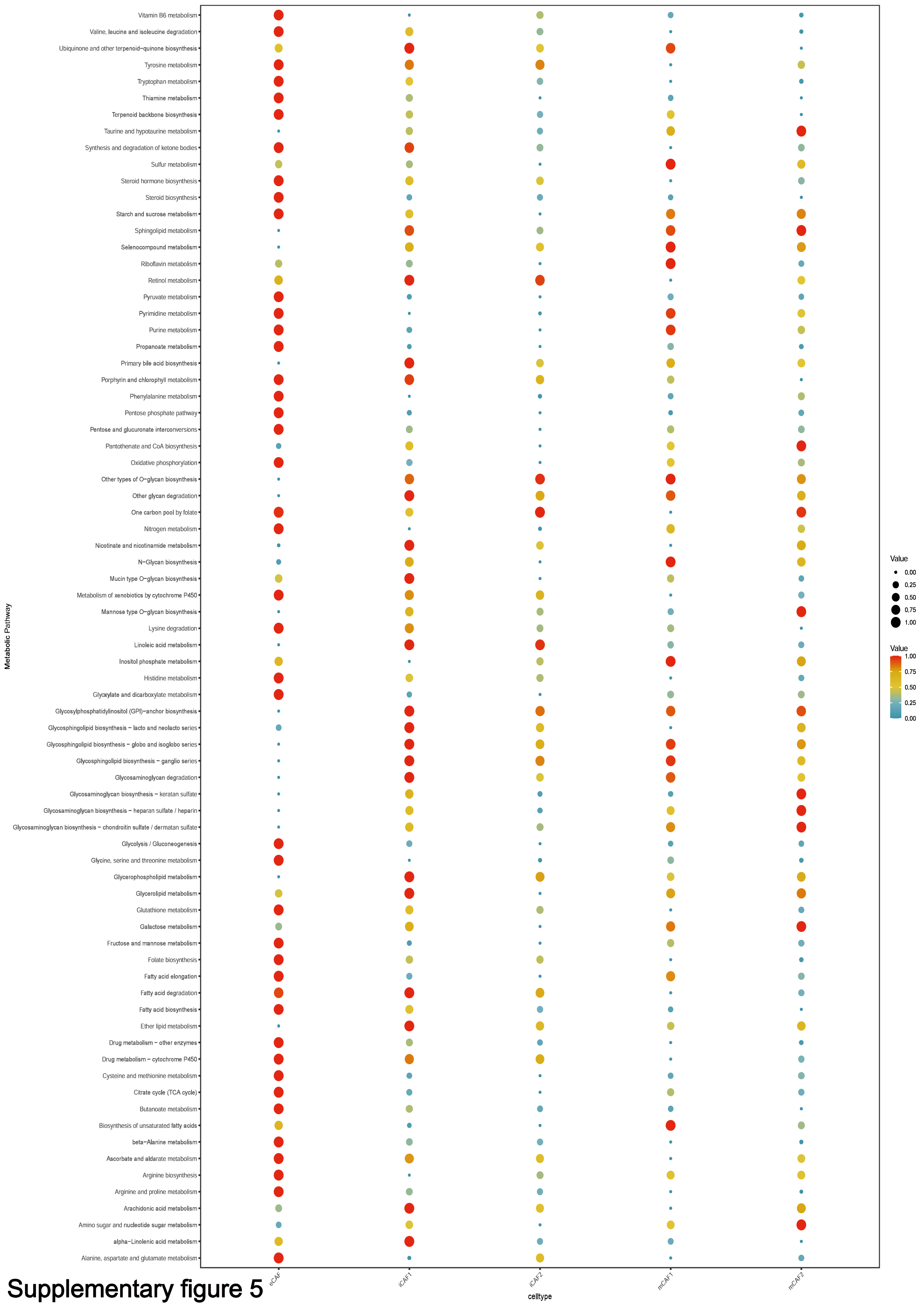

Supplement: Supplementary file 5 — Supplementary Information [file CTM2-13-e1203-s002.tif]
